# Liver Histomorphological Changes in First‐ and Second‐Generation Rat Offspring Associated With Maternal Undernutrition

**DOI:** 10.1155/cjgh/3752573

**Published:** 2025-11-20

**Authors:** Renata Simkunaite-Rizgeliene, Rosita Reivytyte, Viktorija Virbauskyte, Ruta Vosyliute, Violeta Zalgeviciene, Violeta Bartuskiene, Ramune Cepuliene, Janina Tutkuviene

**Affiliations:** ^1^ Department of Anatomy, Histology and Anthropology, Faculty of Medicine, Institute of Biomedical Sciences, Vilnius University, Ciurlionio Street 21, Vilnius, LT-03101, Lithuania, vu.lt; ^2^ Faculty of Medicine, Vilnius University, Ciurlionio Street 21, Vilnius, LT-03101, Lithuania, vu.lt

## Abstract

**Background:**

Due to poverty and the pervasive thin‐body ideal, undernutrition poses a significant challenge in both developed and economically undeveloped nations. Maternal nutritional deprivation has been linked to negative outcomes for the health of the fetus, a higher chance of metabolic syndrome, and adult obesity.

**Aim:**

Considering the functions of the liver, this study aims to assess the interface between maternal malnutrition and morphological changes of the liver in the first and second generations of aged offspring.

**Methods:**

This experimental study conducted in the Department of Anatomy, Histology, and Anthropology involved 26 rats divided into 3 groups: a control group fed a standard diet, a group subjected to 50% dietary restriction before pregnancy, and a group experiencing 50% dietary restriction before and during pregnancy. Histopathological examination was conducted on the livers of both first‐ and second‐generation rat offspring to assess the occurrence of hepatic steatosis, ballooning, inflammation, and fibrosis. Data comparisons were performed using the Kruskal–Wallis test.

**Results:**

Both the first‐ and second‐generation experimental groups displayed a more pronounced steatosis and ballooning index compared to the control group. In addition to this, both male and female progeny of the experimental groups exhibited higher levels of steatosis and ballooning. Offspring of mothers undernourished before pregnancy demonstrated a more severe case of steatosis, whereas offspring from mothers fed a low‐calorie diet before and throughout pregnancy showed a higher ballooning index. In the second generation, these changes were less profound than those seen in the first generation. Notably, both experimental groups of the first generation exhibited significantly higher levels of steatosis compared to their equivalent second‐generation counterparts.

**Conclusions:**

According to this study, there is a link between maternal undernutrition and a higher risk of nonalcoholic steatohepatitis or nonalcoholic fatty liver disease in the offspring’s later life.

## 1. Introduction

Undernutrition is a significant concern in economically underdeveloped countries, as well as the issue of the prevailing slim‐body ideal in well‐developed societies. Research indicates that maternal nutritional deprivation can affect fetal health and growth, leading to insulin resistance, an increased risk of metabolic syndrome, and obesity in adult life [1, 2]. The hypothesis of developmental origins of health and disease (DOHaD) states that health problems in later life arise from a difference between the actual postnatal environment and that expected by the fetus during its developmental phase [3–6]. The physiological alterations observed in offspring, along with disturbances in homeostasis and overall well‐being, may be attributed to changes in tissue morphology that occur during in utero programming processes, wherein the fetus acquires the ability to adapt to early‐life conditions [7–9]. This can be explained by developmental plasticity, which shows how the environment changes during crucial periods of early life and determines the actual development of the organism and the final phenotype [3–5]. This concept is closely aligned with the already mentioned DOHaD theory, which emphasizes that early nutrition has a lasting impact on health throughout life and across generations through epigenetic changes, underscoring the importance of transgenerational research for effective public health strategies [10, 11].

Numerous studies have been conducted in the field of growth programming, investigating how maternal malnutrition influences the developing fetus’s body. Research has been carried out on the lungs, pancreas, thyroid gland, skeletal system, adipose tissue, and reproductive system [12–17]. There are articles addressing the impact of maternal undernutrition on the liver both *in vitro* and *in vivo*, yet insufficiently. Studies *in vitro* showed elevated intracellular triglyceride levels in hepatic cells cultured in amino acid–deficient medium [18]. These findings are supported by evidence from both human famine events and experimental models, which had demonstrated that intrauterine undernutrition can program liver metabolism and promote hepatic fat accumulation in the offspring [19–21]. Notably, previous detailed studies of the livers of malnourished maternal offspring showed histological similarities to nonalcoholic fatty liver disease (NAFLD) (called metabolic dysfunction–associated steatotic liver disease [MASLD]) in rats [22, 23]. Research on the relationship between malnutrition and fatty liver is limited. While much attention is given to overnutrition, undernutrition or nutrient imbalance can be equally detrimental, especially during critical windows of development. Caloric restriction has been shown to impact offspring in both the short and long term. These findings underscore the importance of maternal nutritional status in shaping offspring liver health and metabolic programming, highlighting the need for further clinical and mechanistic studies in this area.

First‐generation offspring have been well studied in literature, primarily under conditions that existed during pregnancy, leaving a notable gap in research concerning second‐generation offspring and preconception conditions. In addition to this, it is crucial to highlight the limited knowledge of histological changes in offspring organs during maternal caloric deprivation not only during but also before pregnancy. Therefore, this study seeks to assess the liver and evaluate histopathological changes in the offspring of mothers predisposed to a low‐calorie diet not only during but also before pregnancy. It aims to examine such changes in both first‐generation and second‐generation offspring as well. Through a comprehensive examination spanning both pregnancy and prepregnancy phases across two generations, this research seeks to show the enduring impact of maternal nutritional status on liver health in subsequent generations and to advance the current understanding of the broader spectrum of outcomes associated with maternal diet.

## 2. Materials and Methods

### 2.1. Experimental Groups

Virgin female Wistar rats (healthy, 10–12 weeks of age, weighing 220 ± 20 g) were randomly divided into three distinct groups:

(1) First experimental group (EG‐I): Rats in this group were subjected to a 50% dietary restriction (10 g feed per day) one month prior to pregnancy. During pregnancy, they were provided with a standard diet.
(2) Second experimental group (EG‐II): Rats in this group were exposed to a 50% dietary restriction (10 g feed per day) one month before and during the pregnancy period.
(3) Control group (CG): Rats in this group were provided with a standard, normal diet throughout the experimental period. The recommended daily allowance of nutrients was 20 g per day.

The feed from *Kiss Py* (Terra Animalis, Kaunas, Lithuania) had a well‐balanced composition, including chips (corn, wheat, and hay), wheat, oats, locust beans, maize, barley, pea flakes, corn flakes, two‐colored sorghum, safflower seeds, sunflower seeds, wheat popcorn, dried carrots, peas, and peanuts. This feed met the nutritional needs of rodents, providing optimal energy value.

All maternal rats were housed individually, while all offspring rats were housed socially in groups of two in clear polycarbonate cages with stainless steel wire lids and hardwood shavings as bedding, under a 12/12‐h artificial light/dark cycle. Room temperature was maintained at 22 ± 1°C, and tap water was available ad libitum throughout the entire experimental period.

### 2.2. Breeding and Sacrifice of Rat Offspring

Upon completion of the dietary intervention, the first‐generation rat offspring born to the rats in all three groups were raised on a standard, normal diet. The first‐generation female offspring were mated at 10–12 weeks of age with male breeders from outside the study. Subsequently, at the age of 20 months, these first‐generation offspring were humanely sacrificed for the experiment. Similarly, second‐generation rat offspring resulting from the mating of the first‐generation offspring were also provided with a standard, unrestricted diet and subjected to sacrifice at the age of 20 months. The age of 20 months was chosen to allow observation of lifelong effects, as it falls between middle and older adulthood in rats [24].

Details of the animal euthanasia method: Rats were deeply anesthetized with a mixture of xylazine (Sedaxylan, Eurovet Animal Health) and ketamine (Ketamidor, Richter Pharma). The animals were then sacrificed by transcardial perfusion, first with 0.9% NaCl solution for 3 min, followed by 15 min with 4% paraformaldehyde in 0.1 M phosphate buffer solution, pH 7.4.

Note: All procedures involving animals in this study were carried out in strict compliance with the ethical guidelines and regulations governing the use of experimental animals to ensure animal welfare and minimize any potential suffering. The animal husbandry and experiments on animals were carried out according to the national and European regulations and were approved by the State Food and Veterinary Service of the Republic of Lithuania (no. G2‐20, 2015).

**Figure 1:**
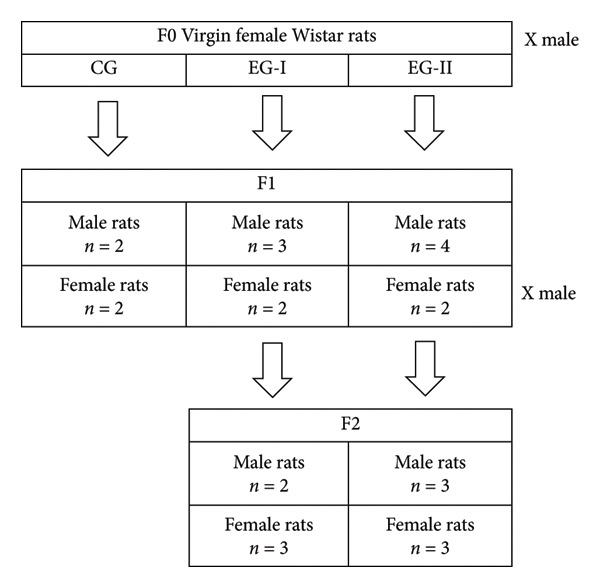
The flowchart of the first‐ and second‐generation rat offspring in this experiment. F0: maternal rats, F1: first generation, F2: second generation, CG: control group, EG‐I: first experimental group, and EG‐II: second experimental group.

A total of 15 rats from the first generation and 11 rats from the second generation were subjects of analysis in the experiment (Figure 1). The sample size was determined based on our previously published data [8] and findings from other researchers [10, 11], aiming to balance the statistical power of the experiment with the ethical consideration of minimizing the number of sacrificed animals. As no CG was established for the second generation, comparisons were made between the second‐generation experimental rats and the first‐generation CG.

### 2.3. Histopathological Examination and Evaluation of Histopathological Changes

Tissue samples were fixed and processed for embedding in paraffin using routine protocols, and 4‐μm‐thick sections were cut using a microtome. Hematoxylin and eosin staining was applied to these sections. The analysis of histopathological changes was conducted with the *CellSens* software tool (Olympus BX43, Germany).

**Figure 2:**
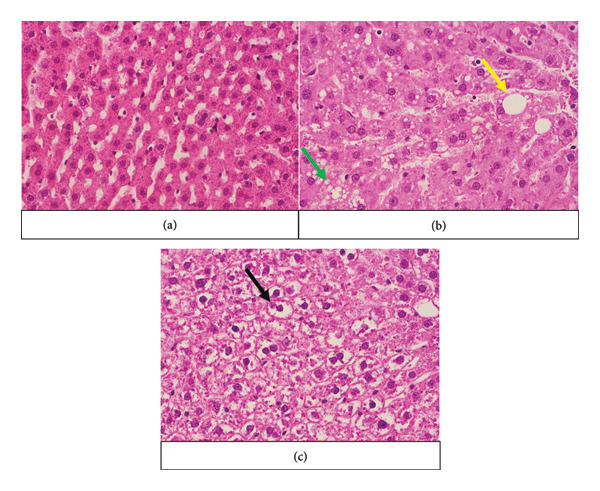
Histopathological changes in the first‐generation male control group and experimental male groups. The yellow arrow shows macrovesicular steatosis, the green arrow shows microvesicular steatosis, and the black arrow shows ballooning degeneration. EG‐I: first experimental group; EG‐II: second experimental group. (a) First‐generation male control group. (b) First‐generation male EG‐I. (c) First‐generation male EG‐II.

**Figure 3:**
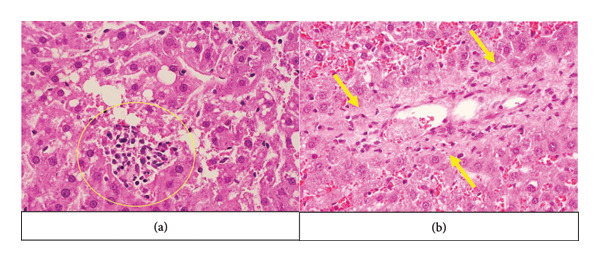
Inflammation and fibrosis in first‐generation male rats of EG‐I and EG‐II. The yellow circle shows the inflammation site, and the yellow arrows point toward the waved collagen fibers (fibrosis). EG‐I: first experimental group and EG‐II: second experimental group. (a) First‐generation male EG‐I. (b) First‐generation male EG‐II.

The evaluation of histopathological changes in the liver sections was carried out following the proposed criteria by Brunt et al. [25]. To assess hepatic steatosis (Figure 2), 10 randomly selected vision fields from each section were examined under 40x magnification. In each of these fields, the surface area of fat droplets was manually marked and calculated with *CellSens* software. Additionally, hepatocyte ballooning (Figure 2) was investigated within the same selected fields, where both normal hepatocytes and ballooned hepatocytes were counted to derive a ballooning index (ratio). Additionally, inflammation and fibrosis in the liver sections were visually evaluated (Figure 3).

### 2.4. Statistical Analysis

All observed histopathological changes were thoroughly documented. Subsequently, comparisons were made between all experimental groups using SPSS Statistics for Windows, Version 29.0 (SPSS Inc., Chicago, Ill., USA) and Microsoft Excel 365 programs. Data comparison was performed using the nonparametric Kruskal–Wallis test with the Bonferroni correction. The difference was considered significant when *p* < 0.05.

## 3. Results

### 3.1. Comparative Analysis of First‐Generation Rat Offspring Histomorphological Changes

#### 3.1.1. The Comparison of Histomorphological Changes by Groups

In both the EG‐I and EG‐II, there was a significant increase in steatosis and ballooning index compared to the CG (*p* < 0.001) (Figure 2) (Table 1). Comparing EG‐I and EG‐II together, it was found that steatosis was more expressed in the EG‐I (*p* < 0.05), whereas the ballooning index showed a greater increase in the EG‐II (*p* < 0.001). No periportal inflammation or fibrosis was visually observed in the CGs. However, all experimental groups exhibited these manifestations (Figure 3), except for the absence of fibrosis in the male EG‐I.

**Table 1:** Comparison by groups in the first generation.

Variables	Control group (*n* = 4)	First experimental group (EG‐I) (*n* = 5)	Second experimental group (EG‐II) (*n* = 6)
Steatosis percentage median (min; max)	0.165 (0.01; 0.93)	6.195 (0.16; 18.61)^a,b^	2.505 (0.45; 11.99)^a,b^
Ballooning index median (min; max)	1.340 (0; 3.7)	6.390 (0; 36.59)^a,c^	25.390 (8.73; 65.54)^a,c^

^a^The difference compared to the control group was found statistically significant (adjusted *p* < 0.001).

^b^The comparison between EG‐I and EG‐II was found statistically significant (adjusted *p* < 0.05).

^c^Comparison between the EG‐I and EG‐II was found statistically significant (adjusted *p* < 0.001).

#### 3.1.2. The Comparison of Histomorphological Changes by Sex

In both EG‐I and EG‐II females and males, steatosis, as well as the ballooning index, showed an increase compared to the CGs (*p* < 0.001), except in the comparison between EG‐I males and the CG, where the increase in the ballooning index was not statistically significant (Table 2). The expression of steatosis in EG‐I males was more pronounced than that in EG‐I females (*p* < 0.05). Additionally, the increase in steatosis among EG‐I males was greater than that in EG‐II males (*p* < 0.001). In contrast, the ballooning index was higher in EG‐II males than in EG‐I males (*p* < 0.001).

**Table 2:** Comparison by sex in the first generation.

Variables	Control group		First experimental group (EG‐I)		Second experimental group (EG‐II)	
	Female (*n* = 2)	Male (*n* = 2)	Female (*n* = 2)	Male (*n* = 3)	Female (*n* = 2)	Male (*n* = 4)
Steatosis percentage median (min; max)	0.065 (0.01; 0.42)	0.345 (0.04; 0.93)	2.650 (0.16; 8.84)^a,d^	7.960 (4.36; 18.61)^b,c,d^	2.155 (0.84; 4.54)^a^	2.635 (0.45; 11.99)^b,c^
Ballooning index median (min; max)	0.825 (0; 2.56)	2.105 (0; 3.7)	12.390 (1.33; 36.59)^a^	4.975 (0; 29.92)^c^	30.225 (15.38; 48.57)^a^	23.670 (8.73; 65.54)^b,c^

^a^The difference compared to the female control group was statistically significant (adjusted *p* < 0.001).

^b^The difference compared to the male control group was statistically significant (adjusted *p* < 0.001).

^c^Comparison between male EG‐I and EG‐II was found statistically significant (adjusted *p* < 0.001).

^d^Comparison between male and female EG‐I was found statistically significant (*p* < 0.05).

### 3.2. Comparative Analysis of Second‐Generation Rat Offspring Histomorphological Changes

#### 3.2.1. The Comparison of Histomorphological Changes by Groups

**Figure 4:**
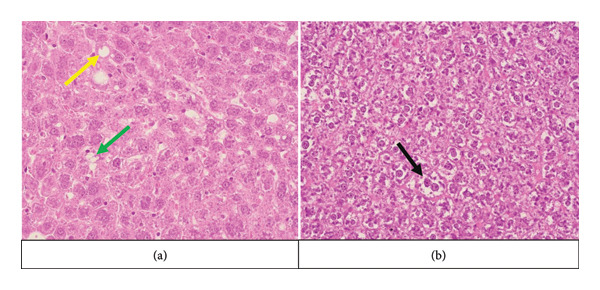
Histopathological changes in second‐generation female experimental groups. The yellow arrow shows macrovesicular steatosis, the green arrow shows microvesicular steatosis, and black arrow shows ballooning degeneration. EG‐I: first experimental group; EG‐II: second experimental group. (a) Second‐generation female EG‐I. (b) Second‐generation female EG‐II.

In the second generation, a similar trend of histopathological changes was observed as in the first generation. Both the EG‐II and the EG‐I showed increased levels of steatosis and the ballooning index compared to the CG (*p* < 0.001) (Figure 4) (Table 3). When comparing EG‐I and EG‐II, it was found that steatosis was more pronounced in the EG‐I (*p* < 0.05), whereas the ballooning index exhibited a greater increase in the EG‐II (*p* < 0.05). Visually evaluated periportal fibrosis and inflammation did not exhibit a specific trend, except for the detection of these changes in all the EG‐I females.

**Table 3:** Comparison by groups in the second generation.

Variables	Control group (*n* = 4)	First experimental group (EG‐I) (*n* = 5)	Second experimental group (EG‐II) (*n* = 6)
Steatosis percentage median (min; max)	0.165 (0.01; 0.93)	3.072 (0.19; 11.04)^a,b^	1.143 (0.06; 10.56)^a,b^
Ballooning index median (min; max)	1.34 (0; 3.7)	8.105 (0; 44.95)^a,b^	20.163 (0; 67.48)^a,b^

^a^The difference compared to the control group was statistically significant (adjusted *p* < 0.001).

^b^Comparison between the EG‐I and EG‐II was found statistically significant (adjusted *p* < 0.05).

#### 3.2.2. The Comparison of Histomorphological Changes by Sex

In both EG‐I and EG‐II females and males, the steatosis and ballooning index were more expressed compared to the CGs (*p* < 0.001), except for the ballooning index in EG‐I males and steatosis in EG‐II males compared to the CG—these changes were not statistically significant (Table 4). Additionally, it was observed that EG‐I males showed a greater increase in steatosis than EG‐II males (*p* < 0.05).

**Table 4:** Comparison by sex in the second generation.

Variables	Control group		First experimental group (EG‐I)		Second experimental group (EG‐II)	
	Female (*n* = 2)	Male (*n* = 2)	Female (*n* = 3)	Male (*n* = 2)	Female (*n* = 3)	Male (*n* = 3)
Steatosis percentage median (min; max)	0.065 (0.01; 0.42)	0.345 (0.04; 0.93)	1.956 (0.19; 11.04)^a^	3.438 (1.16; 7.24)^b,c^	1.984 (0.09; 10.56)^a^	0.374 (0.06; 5.74)^c^
Ballooning index median (min; max)	0.825 (0; 2.56)	2.105 (0; 3.7)	9.620 (2.4; 18.02)^a^	4.398 (0; 44.95)	19.984 (5.69; 67.48)^a^	21.017 (0; 47.62)^b^

^a^The difference compared to the female control group was statistically significant (adjusted *p* < 0.001).

^b^The difference compared to the male control group was statistically significant (adjusted *p* < 0.001).

^c^Comparison between male EG‐I and EG‐II was found statistically significant (adjusted *p* < 0.05).

### 3.3. Comparison of First‐ and Second‐Generation Histomorphological Changes

It was found that steatosis in the first‐generation EG‐I and EG‐II showed a significantly greater increase than that in the corresponding second‐generation EG‐I (*p* < 0.05) and EG‐II (*p* < 0.05) (Table 5). Additionally, it was observed that steatosis was more expressed in the first‐generation EG‐II males than in the second‐generation EG‐II males (*p* < 0.05) (Table 6).

**Table 5:** Comparison by groups in the first and second generations.

Variables	First experimental group (EG‐I)		Second experimental group (EG‐II)	
	1st generation (*n* = 5)	2nd generation (*n* = 5)	1st generation (*n* = 6)	2nd generation (*n* = 6)
Steatosis percentage median (min; max)	6.195 (0.16; 18.61)^a^	3.072 (0.19; 11.04)^a^	2.505 (0.45; 11.99)^b^	1.143 (0.06; 10.56)^b^
Ballooning index median (min; max)	6.390 (0; 36.59)	8.105 (0; 44.95)	25.390 (8.73; 65.54)	20.163 (0; 67.48)

^a^EG‐I comparison between generations was found statistically significant (adjusted *p* < 0.05).

^b^EG‐II comparison between generations was found statistically significant (adjusted *p* < 0.05).

**Table 6:** Comparison by sex in the first and second generations.

Variables	EG‐I female		EG‐II female		EG‐I male		EG‐II male	
	1st gen (*n* = 2)	2nd gen (*n* = 3)	1st gen (*n* = 2)	2nd gen (*n* = 3)	1st gen (*n* = 3)	2nd gen (*n* = 2)	1st gen (*n* = 4)	2nd gen (*n* = 3)
Steatosis percentage median (min; max)	2.650 (0.16; 8.84)	1.956 (0.19; 11.04)	2.155 (0.84; 4.54)	1.984 (0.09; 10.56)	7.960 (4.36; 18.61)	3.438 (1.16; 7.24)	2.635 (0.45; 11.99)^a^	0.374 (0.06; 5.74)^a^
Ballooning index median (min; max)	12.390 (1.33; 36.59)	9.620 (2.4; 18.02)	30.225 (15.38; 48.57)	19.984 (5.69; 67.48)	4.975 (0; 29.92)	4.398 (0; 44.95)	23.670 (8.73; 65.54)	21.017 (0; 47.62)

Abbreviations: EG‐I = first experimental group; EG‐II = second experimental group; gen = generation.

^a^Male EG‐II comparison between generations was found statistically significant (adjusted *p* < 0.05).

## 4. Discussion

Liver biopsy has historically been considered the gold standard for confirming and differentiating between NAFLD and nonalcoholic steatohepatitis (NASH) in clinical practice. Although these conditions are recently referred to as MASLD and metabolic dysfunction–associated steatohepatitis (MASH), the terms NAFLD and NASH remain widely used in older literature and will be used throughout this discussion for consistency. The histological features mentioned—steatosis for NAFLD and additional features like hepatocyte ballooning, inflammation, and fibrosis for NASH—are indeed key criteria used in the diagnosis [22, 26].

This study identified similar histological characteristics, aligning with the definitions mentioned earlier. Other studies have attempted to identify the reasons behind the onset of these diseases. It has been established that maternal undernutrition can contribute to the development of these particular features [18, 21, 27–29]. In order to adapt, embryos undergo “foetal programming”—a term used to describe the energy alterations and metabolism that change to ensure the growth of vital organs (e.g., heart and brain) by reducing energy consumption in nonprimary organs (e.g., liver) [4–7, 17]. Animal studies were not the only approach used to investigate whether maternal undernutrition causes changes in offspring tissue and organ formation. The Dutch cohort, which collected data from the Hunger Winter in 1944, and the Chinese “Great Famine” study also demonstrated that mothers who experienced starvation cause long‐term metabolic changes in their offspring’s liver and increase the likelihood of developing NAFLD [19, 26].

One of the main functions of the liver is to conduct gluconeogenesis and lipogenesis. The offspring of undernourished mothers undergo postnatal suppression of certain hepatic genes, resulting in increased lipogenesis and fat storage and decreased lipolysis [18, 20]. Fat accumulates in the liver tissue as triglycerides so that gluconeogenesis can occur [30]. Consequently, hepatic steatosis develops.

Both excess and deficiency of various maternal dietary components (carbohydrates, proteins, fats, and so on) can program the offspring toward metabolic diseases, exerting lasting effects on liver development and metabolism through distinct mechanisms [31–33]. Although many factors are known to contribute to NAFLD, the mechanisms underlying hepatic steatosis following maternal undernutrition remain largely unclear. The majority of studies investigating the mechanisms of steatosis development focus on the effects of protein restriction. Dietary components such as amino acids act as endocrine factors extracellularly and as intracellular signaling mediators to regulate metabolism [18]. Therefore, dietary protein–mediated signaling can influence metabolic regulation, potentially leading to a NAFLD‐like phenotype.

Severe maternal protein restriction may promote transgenerational metabolic programming through dysregulation of the adipoinsular axis and the development of hypothalamic leptin resistance [10]. It is known that rats fed a low‐protein diet exhibit reduced serum insulin and insulin‐like growth factor I (IGF‐I) levels, along with increased insulin resistance. Some researchers have hypothesized that increased resistance to insulin may contribute to the development of fatty liver [21, 22]. However, some evidence suggests that triglyceride accumulation in the liver may occur independently of insulin signaling upregulation [18].

In addition to these metabolic changes, increased production of reactive oxygen species leads to chronic inflammation, which sustains hepatic steatosis and inflicts damage on hepatocytes [34]. Our study revealed that while steatosis was prominent in both experimental groups, it was more expressed in the rat offspring of preconceptionally undernourished mothers. Conversely, hepatic ballooning was more prevalent in the rat offspring whose mothers were fed a restricted diet before and during pregnancy. This occurrence might be attributed to the fact that during pregnancy, the fetus primarily relies on the mother for its energy supply [3]. Therefore, ballooning of the liver may be a sign of greater liver damage compared to steatosis.

We observed hepatic inflammation and fibrosis in the experimental groups of our study. Observations may be explained by a lack of dietary amino acids that stimulate the secretion of fibroblast growth factor (FGF) 21, known for its role in the development of fibrosis [35]. In addition, inflammatory responses are evident in the offspring of undernourished mothers due to prenatal epigenetic changes that lead to postnatal fat accumulation and inflammation mediated by Kupffer cells or mitochondrial dysfunction [3, 22, 36].

Studies report different sex‐related findings. Studies show that female offspring are more adaptive than male offspring [1, 9, 15, 20]. Furthermore, certain studies propose that females and males develop distinct patterns of strategies in response to maternal undernutrition [37]. Our study identified changes in both first‐generation female and male offspring.

The influence of maternal diet is known to affect not only first‐generation offspring but also second‐generation offspring [10, 11, 13]. There are a limited number of studies analyzing these changes in the second generation, making it difficult to identify a trend. Nevertheless, several studies revealed that second‐generation male offspring had higher body weight. However, as for the retina, some changes were manifested in a positive direction and others in a negative direction [8].

Our research showed that the majority of the observed changes in the liver of the second generation were less pronounced. This phenomenon may be attributed to the liver’s greater plasticity in the context of maternal undernutrition, rendering the liver more adaptable and responsive to changes. Thus, understanding the plasticity of the liver in response to maternal undernutrition is essential for elucidating the long‐term health consequences for offspring.

Since such experiments cannot be conducted on humans, rats are a suitable model because they reproduce quickly, allowing changes to be observed over several generations, and their organ structures closely resemble those of humans.

While this study presents certain limitations, it also offers important contributions to a relatively underexplored field. Firstly, the modest sample size may limit the extent to which the findings can be applied to broader populations. However, given the limited number of existing studies in this area and the ethical considerations regarding animal welfare, the study provides valuable insights that lay the groundwork for future investigations. Secondly, the assessment of fibrosis using histochemistry staining methods was not performed, limiting a comprehensive evaluation of these aspects. Nevertheless, the methodology used in this study is adequate for detecting these histopathological changes. Additionally, the histological analysis was performed manually, which could introduce some variability. Nevertheless, the meticulous analysis of the samples by several researchers enhances the reliability of the findings.

## 5. Conclusions

The findings underscore the enduring impact of maternal nutritional status on the liver health of subsequent generations. The presence of hepatic steatosis, hepatocellular ballooning, and other histopathological alterations suggests a transgenerational influence of maternal undernutrition on the susceptibility to liver‐related conditions in offspring. In addition to this, there might be a link between a mother’s undernutrition and NAFLD or NASH development in offspring. This insight raises important considerations regarding the long‐term consequences of maternal dietary factors on liver health and emphasizes the need for comprehensive interventions and awareness to address potential intergenerational health outcomes. Further research and exploration into the underlying mechanisms of these transgenerational effects would contribute valuable insights to the field of maternal and child health.

## Conflicts of Interest

The authors declare no conflicts of interest.

## Funding

No funding was received for this study.

## Data Availability

The data that support the findings of this study are available from the corresponding author upon reasonable request.

## References

[1] Bellinger L. and Langley-Evans S. C. , Fetal Programming of Appetite by Exposure to a Maternal Low-Protein Diet in the Rat, Clinical Science. (September 2005) 109, no. 4, 413–420, 10.1042/CS20050127, 2-s2.0-26844503967. 15992360

[2] Ramadan W. S. , Alshiraihi I. , and Al-karim S. , Effect of Maternal Low Protein Diet During Pregnancy on the Fetal Liver of Rats, Annals of Anatomy. (2013) 195, no. 1, 68–76, 10.1016/j.aanat.2012.05.006, 2-s2.0-84873085559. 22877887

[3] Morris T. J. , Vickers M. , Gluckman P. , Gilmour S. , and Affara N. , Transcriptional Profiling of Rats Subjected to Gestational Undernourishment: Implications for the Developmental Variations in Metabolic Traits, PLoS One. (September 2009) 4, no. 9, 10.1371/journal.pone.0007271, 2-s2.0-70349668913. PMC2749934 19787071

[4] Gluckman P. D. , Hanson M. A. , and Pinal C. , The Developmental Origins of Adult Disease, Maternal and Child Nutrition. (2005) 1, no. 3, 130–141, 10.1111/j.1740-8709.2005.00020.x, 2-s2.0-28244472326. 16881892 PMC6860944

[5] Gluckman P. D. , Hanson M. A. , Morton S. M. B. , and Pinal C. S. , Life-Long Echoes – A Critical Analysis of the Developmental Origins of Adult Disease Model, Neonatology. (2005) 87, no. 2, 127–139, 10.1159/000082311, 2-s2.0-14044279827. 15564779

[6] Hoffman D. J. , Powell T. L. , Barrett E. S. , and Hardy D. B. , Developmental Origins of Metabolic Diseases, Physiological Reviews. (July 2021) 101, no. 3, 739–795, 10.1152/physrev.00002.2020. 33270534 PMC8526339

[7] Langley-Evans S. C. , Developmental Programming of Health and Disease, Proceedings of the Nutrition Society. (February 2006) 65, no. 1, 97–105, 10.1079/pns2005478, 2-s2.0-32944457515. 16441949 PMC1885472

[8] Laurinaviciute G. , Simkunaite-Rizgeliene R. , Zalgeviciene V. et al., Maternal Undernutrition Model of Two Generations of Rats: Changes in the Aged Retina, Histology & Histopathology. (April 2023) 38, no. 4, 409–422, 10.14670/HH-18-522. 36148876

[9] Araminaite V. , Zalgeviciene V. , Simkunaite-Rizgeliene R. , Stukas R. , Kaminskas A. , and Tutkuviene J. , Maternal Caloric Restriction Prior to Pregnancy Increases the Body Weight of the Second-Generation Male Offspring and Shortens Their Longevity in Rats, Tohoku Journal of Experimental Medicine. (August 2014) 234, no. 1, 41–50, 10.1620/tjem.234.41, 2-s2.0-84919383483. 25175031

[10] Peixoto-Silva N. , Frantz E. D. , Mandarim-de-Lacerda C. A. , and Pinheiro-Mulder A. , Maternal Protein Restriction in Mice Causes Adverse Metabolic and Hypothalamic Effects in the F1 and F2 Generations, British Journal of Nutrition. (2011) 106, no. 9, 1364–1373, 10.1017/S0007114511001735, 2-s2.0-80055061449. 21736811

[11] Harrison M. and Langley-Evans S. C. , Intergenerational Programming of Impaired Nephrogenesis and Hypertension in Rats Following Maternal Protein Restriction During Pregnancy, British Journal of Nutrition. (2008) 101, no. 7, 1020–1030, 10.1017/S0007114508057607, 2-s2.0-67449102553. 18778527 PMC2665257

[12] Fandiño J. , Toba L. , González-Matías L. C. , Diz-Chaves Y. , and Mallo F. , Perinatal Undernutrition, Metabolic Hormones, and Lung Development, Nutrients. (November 2019) 11, no. 12, 10.3390/nu11122870. PMC6950278 31771174

[13] Frantz E. D. C. , Aguila M. B. , Pinheiro-Mulder A. d. R. , and Mandarim-de-Lacerda C. A. , Transgenerational Endocrine Pancreatic Adaptation in Mice From Maternal Protein Restriction in Utero, Mechanism of Ageing and Development. (March 2011) 132, no. 3, 110–116, 10.1016/j.mad.2011.01.003, 2-s2.0-79952774174. 21291904

[14] Miranda R. A. , de Moura E. G. , and Lisboa P. C. , Adverse Perinatal Conditions and the Developmental Origins of Thyroid Dysfunction – Lessons From Animal Models, Endocrine. (August 2022) 79, no. 2, 223–234, 10.1007/s12020-022-03177-7. 36036880

[15] Lukaszewski M. A. , Eberlé D. , Vieau D. , and Breton C. , Nutritional Manipulations in the Perinatal Period Program Adipose Tissue in Offspring, American Journal of Physiology-Endocrinology and Metabolism. (November 2013) 305, no. 10, E1195–E1207, 10.1152/ajpendo.00231.2013, 2-s2.0-84887589406. 24045869

[16] Hoffman F. , Boretto E. , Vitale S. et al., Maternal Nutritional Restriction During Late Gestation Impairs Development of the Reproductive Organs in Both Male and Female Lambs, Theriogenology. (March 2018) 108, 331–338, 10.1016/j.theriogenology.2017.12.023, 2-s2.0-85039420604. 29288977

[17] Castrogiovanni P. and Imbesi R. , The Role of Malnutrition During Pregnancy and Its Effects on Brain and Skeletal Muscle Postnatal Development, Journal of Functional Morphology and Kinesiology. (August 2017) 2, no. 3, 10.3390/jfmk2030030, 2-s2.0-85062618969.

[18] Nishi H. , Yamanaka D. , Kamei H. et al., Importance of Serum Amino Acid Profile for Induction of Hepatic Steatosis Under Protein Malnutrition, Scientific Reports. (April 2018) 8, no. 1, 10.1038/s41598-018-23640-8, 2-s2.0-85044978774. PMC5882898 29615653

[19] Zheng X. , Ren W. , Gong L. , Long J. , Luo R. , and Wang Y. , The Great Chinese Famine Exposure in Early Life and the Risk of Nonalcoholic Fatty Liver Disease in Adult Women, Annals of Hepatology. (November 2017) 16, no. 6, 901–908, 10.5604/01.3001.0010.5281, 2-s2.0-85031896309. 29055916

[20] Zhou X. , Yang H. , Yan Q. et al., Evidence for Liver Energy Metabolism Programming in Offspring Subjected to Intrauterine Undernutrition During Midgestation, Nutrition and Metabolism. (December 2019) 16, no. 1, 10.1186/s12986-019-0346-7, 2-s2.0-85063162069. PMC6423887 30923555

[21] Guéant J. L. , Elakoum R. , Ziegler O. et al., Nutritional Models of Foetal Programming and Nutrigenomic and Epigenomic Dysregulations of Fatty Acid Metabolism in the Liver and Heart, Pfluegers Archiv European Journal of Physiology. (2014) 466, no. 5, 833–850, 10.1007/s00424-013-1339-4, 2-s2.0-84901925031. 23999818

[22] Kucera O. and Cervinkova Z. , Experimental Models of Non-Alcoholic Fatty Liver Disease in Rats, World Journal of Gastroenterology. (2014) 20, no. 26, 8364–8376, 10.3748/wjg.v20.i26.8364, 2-s2.0-84904396612. 25024595 PMC4093690

[23] Du J. E. , You Y. A. , Kwon E. J. et al., Maternal Malnutrition Affects Hepatic Metabolism Through Decreased Hepatic Taurine Levels and Changes in hnf4a Methylation, International Journal of Molecular Sciences. (December 2020) 21, no. 23, 10.3390/ijms21239060. PMC7729756 33260590

[24] Ghasemi A. , Jeddi S. , and Kashfi K. , The Laboratory Rat: Age and Body Weight Matter, EXCLI Journal. (2021) 20, 1431–1445, 10.17179/excli2021-4072. 34737685 PMC8564917

[25] Brunt E. M. , Janney C. G. , Di Bisceglie A. M. , Neuschwander-Tetri B. A. , and Bacon B. R. , Nonalcoholic Steatohepatitis: A Proposal for Grading and Staging the Histological Lesions, American Journal of Gastroenterology. (1999) 94, no. 9, 2467–2474, 10.1111/j.1572-0241.1999.01377.x, 2-s2.0-0033200054. 10484010

[26] Abenavoli L. , Influence of Famine in Women With Non-Alcoholic Fatty Liver Disease, Annals of Hepatology. (2017) 16, no. 6, 826–827, 10.5604/01.3001.0010.5269, 2-s2.0-85031914098. 29055912

[27] Muramatsu-Kato K. , Itoh H. , Kohmura-Kobayashi Y. et al., Undernourishment in Utero Primes Hepatic Steatosis in Adult Mice Offspring on an Obesogenic Diet; Involvement of Endoplasmic Reticulum Stress, Scientific Reports. (November 2015) 5, no. 1, 10.1038/srep16867, 2-s2.0-84947779847. PMC4652266 26581663

[28] Quek S. X. Z. , Tan E. X. X. , Ren Y. P. et al., Factors Early in Life Associated With Hepatic Steatosis, World Journal of Hepatology. (June 2022) 14, no. 6, 1235–1247, 10.4254/wjh.v14.i6.1235. 35978672 PMC9258263

[29] Campisano S. E. , Echarte S. M. , Podaza E. , and Chisari A. N. , Protein Malnutrition During Fetal Programming Induces Fatty Liver in Adult Male Offspring Rats, Journal of Physiology & Biochemistry. (May 2017) 73, no. 2, 275–285, 10.1007/s13105-017-0549-1, 2-s2.0-85011632962. 28160259

[30] García-García R. M. , Arias-álvarez M. , Millán P. et al., Gestation Food Restriction and Refeeding Compensate Maternal Energy Status and Alleviate Metabolic Consequences in Juvenile Offspring in a Rabbit Model, Nutrients. (February 2021) 13, no. 2, 1–18, 10.3390/nu13020310. PMC7912334 33499108

[31] Xue L. , Chen X. , Sun J. et al., Maternal Dietary Carbohydrate and Pregnancy Outcomes: Quality Over Quantity, Nutrients. (2024) 16, no. 14, 10.3390/nu16142269. PMC11280101 39064712

[32] Martin L. J. , Meng Q. , Blencowe M. et al., Maternal High-Protein and Low-Protein Diets Perturb Hypothalamus and Liver Transcriptome and Metabolic Homeostasis in Adult Mouse Offspring, Frontiers in Genetics. (2018) 9, 10.3389/fgene.2018.00642. PMC6297185 30619467

[33] Bowman C. E. , Selen Alpergin E. S. , Cavagnini K. , Smith D. M. , Scafidi S. , and Wolfgang M. J. , Maternal Lipid Metabolism Directs Fetal Liver Programming Following Nutrient Stress, Cell Reports. (2019) 29, no. 5, 1299–1310.e3, 10.1016/j.celrep.2019.09.053, 2-s2.0-85073748589. 31665641 PMC6896898

[34] Lecoutre S. , Montel V. , Vallez E. et al., Transcription Profiling in the Liver of Undernourished Male Rat Offspring Reveals Altered Lipid Metabolism Pathways and Predisposition to Hepatic Steatosis, American Journal of Physiology-Endocrinology and Metabolism. (2019) 317, no. 6, 1094–1107, 10.1152/ajpendo.00291.2019. 31638854

[35] Falamarzi K. , Malekpour M. , Tafti M. F. , Azarpira N. , Behboodi M. , and Zarei M. , The Role of FGF21 and Its Analogs on Liver Associated Diseases, Frontiers of Medicine. (November 2022) 9, 10.3389/fmed.2022.967375. PMC9705724 36457562

[36] Bruce K. D. and Byrne C. D. , The Metabolic Syndrome: Common Origins of a Multifactorial Disorder, Postgraduate Medical Journal. (2009) 85, no. 1009, 614–621, 10.1136/pgmj.2008.078014, 2-s2.0-73249128357. 19892897

[37] Poore K. R. , Hollis L. J. , Murray R. J. S. et al., Differential Pathways to Adult Metabolic Dysfunction Following Poor Nutrition at Two Critical Developmental Periods in Sheep, PLoS One. (March 2014) 9, no. 3, 10.1371/journal.pone.0090994, 2-s2.0-84897442188. PMC3946277 24603546

